# Climate Change and Children’s Health: Protecting and Preparing Our Youngest

**DOI:** 10.1289/ehp.119-a132b

**Published:** 2011-03

**Authors:** Tanya Tillett

**Affiliations:** **Tanya Tillett**, MA, of Durham, NC, is a staff writer/editor for *EHP*. She has been on the *EHP* staff since 2000 and has represented the journal at national and international conferences

Climate change is expected to bring increased frequency and intensity of rainstorms, snowstorms, heat waves, and other extreme weather events. Numerous studies indicate climate change is already contributing to a greater overall burden of disease. A new review uses a children’s health framework to summarize the latest data on the projected increasing burden of climate change–related disease for children **[*EHP*** **119(3):291–298; Sheffield and Landrigan].** The authors also discuss prevention strategies they believe should be incorporated into public health programs.

For 2000 the World Health Organization (WHO) estimated climate change contributed to more than 150,000 deaths and 5.5 million lost disability-adjusted life years worldwide. More than 88% of this burden occurs in children under age 5 years, even though the overall pediatric burden of disease is only 5% in high-income countries and 31% in low- and medium-income countries.

Children are inherently sensitive to the climate because they are physiologically and metabolically less effective than adults at adapting to heat and other climate-related exposures. Rapid development and higher exposures per unit of body weight make them more vulnerable to environmental exposures, and their diet and behavior may expose them to different agents than adults might typically encounter. More expected future years of life provides more time for exposure to new or worsening hazards, and a dependence on caregivers means children can’t always control their surroundings or remove themselves from harm.

In the current review, the authors analyzed health outcomes expected to result from increased temperatures, increasing frequency and severity of extreme weather, and sea-level rise. These include higher rates of vectorborne diseases such as malaria and dengue and of diarrheal disease, more exposure to extreme weather and to toxic chemicals (for instance, as weather changes affect patterns of pesticide use), and greater risks of poverty and of displacement due to sea-level rise, crop failure, and food insecurity. Other impacts include malnutrition and problems related to increased exposures to allergens and air pollution. Risk varies across socioeconomic levels and geographic locations.

The authors write that prevention strategies to help alleviate children’s burden of disease should incorporate climate change adaptation into current programs as well as monitor children’s exposures and environmental health indicators in a manner that is internationally consistent—as proposed by the WHO. They emphasize that new climate-sensitive disease-prevention programs should strive not only to protect children and parents in the short term but also prepare children to be resilient adults in the years to come. They also point to health impact assessments as an emerging tool to help shape smart policies that can solve multiple existing problems and head off future burdens.

